# Macaque Monkeys Can Learn Token Values from Human Models through Vicarious Reward

**DOI:** 10.1371/journal.pone.0059961

**Published:** 2013-03-27

**Authors:** Sara Bevacqua, Erika Cerasti, Rossella Falcone, Milena Cervelloni, Emiliano Brunamonti, Stefano Ferraina, Aldo Genovesio

**Affiliations:** Department of Physiology and Pharmacology, Sapienza University of Rome, Rome, Italy; Université Pierre et Marie Curie, France

## Abstract

Monkeys can learn the symbolic meaning of tokens, and exchange them to get a reward. Monkeys can also learn the symbolic value of a token by observing conspecifics but it is not clear if they can learn passively by observing other actors, e.g., humans. To answer this question, we tested two monkeys in a token exchange paradigm in three experiments. Monkeys learned token values through observation of human models exchanging them. We used, after a phase of object familiarization, different sets of tokens. One token of each set was rewarded with a bit of apple. Other tokens had zero value (neutral tokens). Each token was presented only in one set. During the observation phase, monkeys watched the human model exchange tokens and watched them consume rewards (vicarious rewards). In the test phase, the monkeys were asked to exchange one of the tokens for food reward. Sets of three tokens were used in the first experiment and sets of two tokens were used in the second and third experiments. The valuable token was presented with different probabilities in the observation phase during the first and second experiments in which the monkeys exchanged the valuable token more frequently than any of the neutral tokens. The third experiments examined the effect of unequal probabilities. Our results support the view that monkeys can learn from non-conspecific actors through vicarious reward, even a symbolic task like the token-exchange task.

## Introduction

Since the first work on chimpanzees [1], [2], a number of studies have examined the ability of non-human primates to exchange tokens for foods, in capuchin [3], [4] and chimpanzees [5], or to obtain either food or tools, in capuchins [6]. In token paradigms, tokens are referred as objects with a symbolic meaning and no intrinsic value. Objects can become tokens when they acquire some value through arbitrary associations made with what is returned in exchange. The initial study in capuchins [3] showed that only females were able to use, and only to a certain degree, different tokens for obtaining low- and high-value food. A more recent study showed that some capuchins understood the association between tokens and quantities of food [7]. This later result represents evidence that the symbolic meaning of a token can go beyond its simple association with the reward. Some individual chimpanzee and capuchin monkeys were able to represent the value corresponding to the sum of different tokens [8]. However, limitations in both the understanding and use of tokens have been reported in several experimental paradigms, either when tokens were not used by monkeys to obtain a reward [9] or when monkeys failed to plan the token’s use ahead to maximize future rewards [10].

Token exchange paradigms have also been examined using observation learning procedures. Learning by observation is an adaptive animal ability, present not only in primates but also in many other species such as mice [11], reptiles [12], fishes [13] and even insects [14]. Its adaptive value may be to shortcut problem solving by avoiding or reducing the time otherwise needed for trial and error learning. In the context of the token exchange learning, it has been shown that the tokens’ value can be learned, not only through direct experience, but also through observation of conspecifics exchanging them [4]. However, in their original experiment, Brosnan and de Waal [4] showed that learning the tokens’ value through observation was limited to the observation of conspecifics. Our previous study [15] challenged this idea in a task less abstract than the token exchange task. We showed [15] that monkeys were able to learn object-reward associations from human models when the monkeys were rewarded vicariously. The introduction of the vicarious reward into the learning paradigm may explain the difference in results between Falcone et al. [15] and Menieur et al. [16]. The latter reported a failure to learn through observation. That study used a concurrent discrimination paradigm, where monkeys observed a human model instead of a conspecific. The study by Brosnan and de Waal [4] did not involve observation of food consumption by the human model (vicarious reward). We decided to test whether introducing the vicarious reward would promote learning in a token exchange paradigm, and thus allow learning from the actions of a non-conspecific. In one of the two monkeys, we controlled for stimulus enhancement effects as a potential explanation of the observational learning [17], [18].

## Methods

### Animals

Animal care, housing and experimental procedures conformed to the European (Directive 86/609/ECC) and Italian (D.L. 116/92) laws on the use of non-human primates in scientific research. The research protocol was approved by the Italian Health Ministry (central Direction for Veterinary Service, approval number 199/2009-B). The housing conditions and the experimental procedures were in accordance with the European law of humane care and use of laboratory animals and complied with the recommendations of the Weatherall report (for the use of non human primates in research). The monkeys were kept in the animal facility of the Department of Physiology and Pharmacology of the University La Sapienza of Rome in a room with ventilation, temperature and air conditioning control in cages produced by Tecniplast (Italy).

Two male rhesus monkeys (macaca mulatta) participated in this study. Animals were tested usually five days per week. The monkeys were on water restriction. On test days, the animals received water during the experiment. Both monkeys were provided with food and supplementary fruit, daily. Water was supplemented after the experimental session, when necessary.

The health of each animal was monitored daily by researchers, the animal care staff and, once a week, by the veterinarian. To enrich their cognitive life, we often introduced new toys into the home cage. Some toys contained food items, which promoted their exploratory behavior. However, the token exchange paradigm was, in and of itself, enrichment. It was an opportunity to build relationships with the human experimenters and thus reduce the potential for stress. To increase the enrichment in the animal housing room, a monitor inside displayed motion pictures. Except as noted, the observation and testing phases of the experiments took place in the housing room with the experimenters standing in front of the animal cage. The housing room included six cages, and the monkeys were paired except during the experimental session.

### Exchange Paradigm

We performed three token-exchange experiments after an initial token pre-training period. Each experiment was separated into two phases: an observation-learning phase and a test phase. The pre-training and the observation-learning phases were in many ways similar to that described by Brosnan and De Waal [3].

Each object used as a token was assigned to only one set, and it was never presented to the animals before the experiment. The tokens were objects of different colors and shapes, all equally easy to grasp, to avoid choices based on the ease of handling. We used objects such as a little container, a pen, or a chewing gum box. We avoided objects that could be dangerous to the monkeys, either physically or through ingestion. We tried, as much as practical, to use objects that were “emotionally” comparable as tokens. We discovered during the familiarization period (see below) that some objects were a priori rejected or ignored by each individual monkey. In order to identify and exclude such objects from our sets, we introduced a preliminary object-evaluation phase. Only objects explored by the monkey through manipulation passed this preliminary screening and were included in a token set. We believe that this screening method served our purpose, although we recognize that a “token preference test”, as the one used by de Wall [3], could have been more reliable. The reason for our choice was to reduce the duration of the daily sessions that, if too long, could have produced a drop of attention in the monkeys. However, it is still possible that our screening method failed in identify some of the non-preferred objects.

Hereafter, we refer to the token associated with the bit of apple as the “valuable token” and to any other token as a “neutral token”.

### Token Familiarization

Before the tokens pre-training, monkeys had to learn to exchange a token. We taught the tokens exchange by progressively increasing the task requirements, such as the precision of the monkey’s exchange. We can loosely divide the different familiarization phase into five steps, although in practice we moved back and forth through them depending on performance. At the beginning, the experimenter stood in front of the monkey with only one token placed in a metallic tray. First step: the monkey was rewarded with a piece of apple just for touching the object. Second step: the monkey was not only required to touch, but also to grasp the object to receive the reward. Third step: the monkey was required to grasp and raise the object. Fourth step: the monkey had to move the object toward the experimenter’s hand. In the fifth and last familiarization step, the monkey was rewarded only when the object was delivered precisely into the experimenter’s hand. All the tokens used in this phase were associated with receiving a reward (valuable tokens) and they were never used again in the three experiments.

### Token Pre-training

In this preliminary phase and for the first few days of the experiment 1, monkeys were trained in an experimental room, while they were sitting on a primate chair and not in the animal room. The reason was that each day, before the pre-training, they participated also in another experiment, a nonmatch-to-goal task [19] not involving tokens. The pre-training was done only once before the experiment 1. Monkeys were pre-trained in the token exchange paradigm with sets of three tokens, one valuable and two neutral, and their performance was video recorded. The recording camera was located on a metallic table placed in front of the animal. Because the tokens used for testing were preselected after an object-evaluation phase, they could differ between monkeys. The tokens were presented on a metallic tray, the same tray used in all three experiments. The tray was moved in proximity of the monkey’s chair at the start of a trial. Once the animal chose an object, the tray was moved away from the monkey to reposition the tokens before the subsequent trial. The same three tokens were presented until the valuable token was exchanged 80% of the time, within a maximum of 20 trials. Both monkeys gradually improved their performance through several days of pre-training. The token pre-training had the main objective to teach the monkeys that only one token could be associated with reward. All tokens used in this phase were discarded from both the observation learning and test phases.

We calculated the percentage of correct trials after the monkeys exchanged the correct token the first time. Monkey 1 performed at 53% (51/96), 60% (15/25), 60% (25/42) and 72% (13/18) in the first four days, reaching criterion the fifth day at 82% (14/17).

Monkey 2 performed at 60% (18/30), 67% (40/60), 67% (16/24), 77% (33/43), and 63% (20/32) the first five days, reaching criterion at 100% (10/10) the sixth day.

### Observation Learning Phase

In this phase, the monkeys could learn token value by watching an experimenter, referred to here as the “human model.” The human model exchanged tokens with another experimenter that provided the food, referred to as “the distributor”. The experimenter playing the role of the distributor was always the same in both the observation learning and the test phases.

**Figure 1:**
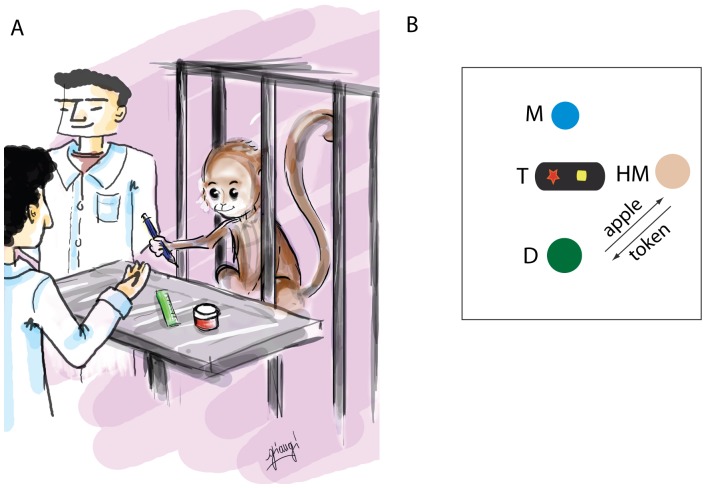
Exchange paradigm. **A.** The tray with tokens (in this case three different tokens) is located in front of the animal’s cage. The distributor (the human in front of the monkey with whom both the monkey and the human model exchange tokens) is in front of the animal with the hand outstretched in a requesting gesture. The human model is standing on the animal’s rightside. Illustration by Angelica Marilungo. **B.** Schematic representation of the apparatus. M: monkey; T: tray with two tokens, D: distributor, HM: human model. Arrows represent an exchange during the observation phase. The human model gives the token to the distributor and the distributor gives the apple in exchange.

During each session (except for the first four days during which the animals were seated on a monkey chair), monkeys were in their cage with the distributor in front of them and the human model standing nearby. The distributor stood in front of the monkey, beyond its reach, showing a metal tray equipped with tokens. The human model stood on the right side of the distributor (Fig.1). In the following, we will refer to a token exchange performed by the human model in front of the monkey as a “token presentation”.

We adopted different levels of difficulty for the learning phase in the three experiments. We asked the monkeys to learn from (1) three tokens with unequal and equal presentation in experiment 1; (2) two tokens with unequal presentation in experiment 2; and (3) two tokens with equal presentation in experiment 3. Extreme care was taken by the experimenter not to cue the monkey as to the location of the valuable token. During the observation phase, the experimenter always gazed at the monkey. Also, the human model, after exchanging the token with the experimenter, looked at the animal. We changed the position of each token one at a time. For example, if the tokens A, B and C were respectively on the right, center and left, and the new assigned positions were A left, B center and C right, we moved token A to the left, and all the other tokens’ positions were changed accordingly, maintaining all the tokens visible.

### Test Phase

In the test phase, in each session, we used a set of either three (experiment 1) or two tokens (experiment 2 and 3). Tokens were placed on a metallic tray and presented to the monkey. For each presentation of a token set (referred as a trial), the monkey was required to choose only one token from the tray. For the first trial the tokens were positioned on the tray by the experimenter and in the other trials of the same session, we used the same procedure as explained for the observation phase. The human model was always on the right side of the experimenter. In all the experiments, the experimenter was standing in front of the monkey, with the left hand outstretched in a requesting gesture, encouraging the token’s return. The human model, that in this phase played no direct role, remained in the room at the same location as described for the observation phase. Monkeys were required to choose a token and to place it into the palm of the experimenter. Throwing the token was an invalid exchange and was not included in the evaluation of the performance. After the chosen token had been returned, the experimenter held it on her left hand palm, to be visible to the monkey. In the meantime, with the right hand, the experimenter took a piece of apple from her pocket and gave it to the animal if the token returned was the valuable one, otherwise, the experimenter presented an empty right hand palm to the monkey. After the *test phase* had been completed for a given set of tokens, a new set was introduced. Between each test phase, the animal was tested in the object-evaluation phase and only for this evaluation without any video recording. All sessions were recorded with a camera and analyzed offline. The camera was located on the left side of the experimenter on a metallic tray. The position of the camera was the same in both the observation the test phases. Trials followed one to another with no interruption. The test phase followed the observation phase after less than a minute. In this period, the experimenter stopped the recording of the observation phase and started recording a new video for the test phase.

### Experiment 1: Three Tokens with Equal and Unequal Presentation of Neutral Tokens

We conducted 31 sessions per monkey, each session consisting of a block of trials of the observation phase followed by a block of four trials in the test phase. For each session, we used a novel set of objects as the tokens, which were fixed across trials within a session. In the *observation phase* of the first four testing days (corresponding to 8 sessions in Monkey 1 and 10 in Monkey 2), the human model grasped and exchanged each token three times, for a total of nine exchanges (presentations). After these initial four days and for all the remaining sessions, the human model made five unequal presentations of the tokens in each session: the human model exchanged the valuable tokens three times and each neutral token one time, for a total of five exchanges. We chose to modify the initial presentation schedule for several reasons, but mainly because we observed a major drop in the monkey’s attention in the last part of the observation phase. In order to prevent the decrease in attention during each daily session, we decided to reduce the total number of neutral tokens presentations. We showed the exchange of the neutral tokens at least once to assure that the monkey experienced the null value associated with them. We reasoned that not establishing a value for neutral tokens might elicit an explorative behavior from the monkey. Further, it has been shown that monkeys and humans perform better during social learning from a negative outcome than from a positive one [20].

Immediately after the observation phase, monkeys were tested with the same set of tokens and the same object as the valuable token in the *observation phase*. Each test phase consisted of a series of four trials. In each trial the position of the valuable token was assigned either to the left, right or middle position of the tray by the experimenter. We did not maintain a fixed location for the valuable token to prevent location enhancement effects.

After token selection the experimenter started recording again and a new observation phase started. The monkeys performed from three to five observation-test sessions per day. The number of sessions per day was decided evaluating the general level of attention and interest of the animal. Often, animal 2 was more attentive and performed 5 sessions per day, while animal 1 lost interest more quickly, limiting the latter to three or four sessions a day.

### Experiment 2: Two Tokens with Unequal Presentation

In this experiment, carried out just after the experiment 1 was ended, we reduced the available choices from three to two. We wanted to test whether part of the difficulty observed in the experiment 1 (especially in monkey 1), depended on the task requirement to monitor the value associated with a great number of tokens. All testing procedures were identical to those used in the experiment 1, except that the test phase consisted of a series of three instead of four trials. The valuable token was exchanged three times and the neutral token one time, as in the previous experiment, for a total of four exchanges. Monkey 1 and monkey 2 were both tested in 50 sessions.

### Experiment 3: Two Tokens with Equal Presentation of Valuable and Null Objects

After we completed the experiment 2, we performed a third experiment with an equal presentation of two tokens. When we decided to perform this experiment Monkey 1 was already starting to participate to another experiment and we decided to test only monkey 2. In this experiment, monkey 2 was tested four times as in experiment one. The goal was to assess whether the learning observed in both previous experiments was affected by the higher frequency of presentation of the valuable token. Presenting the valuable token more frequently could have produced a type of social learning referred as *stimulus enhancement* [21], for which the social learning would result from a greater exposure to the valuable token. To control for the influence of a potential *stimulus enhancement* effect in monkey 2, we tested its ability to learn by observation with an equal number of presentations of the valuable and neutral tokens. In this experiment, the monkey observed the human model exchanging each token two times. Monkey 2 was tested for 30 sessions.

## Results

### Behaviour during Observation Learning

During the observation phase, monkey 2 was very attentive, looking at the human model’s action and waiting for its turn. In contrast, monkey 1 was more restless in the cage, extending arms toward the human model as if trying to obtain the food and showing a greater difficulty with paying attention to the task.

### Experiment 1

In the following analysis we included the results of experiment 1, consisting of sessions with equal presentation and unequal presentation (remaining sessions).

**Figure 2:**
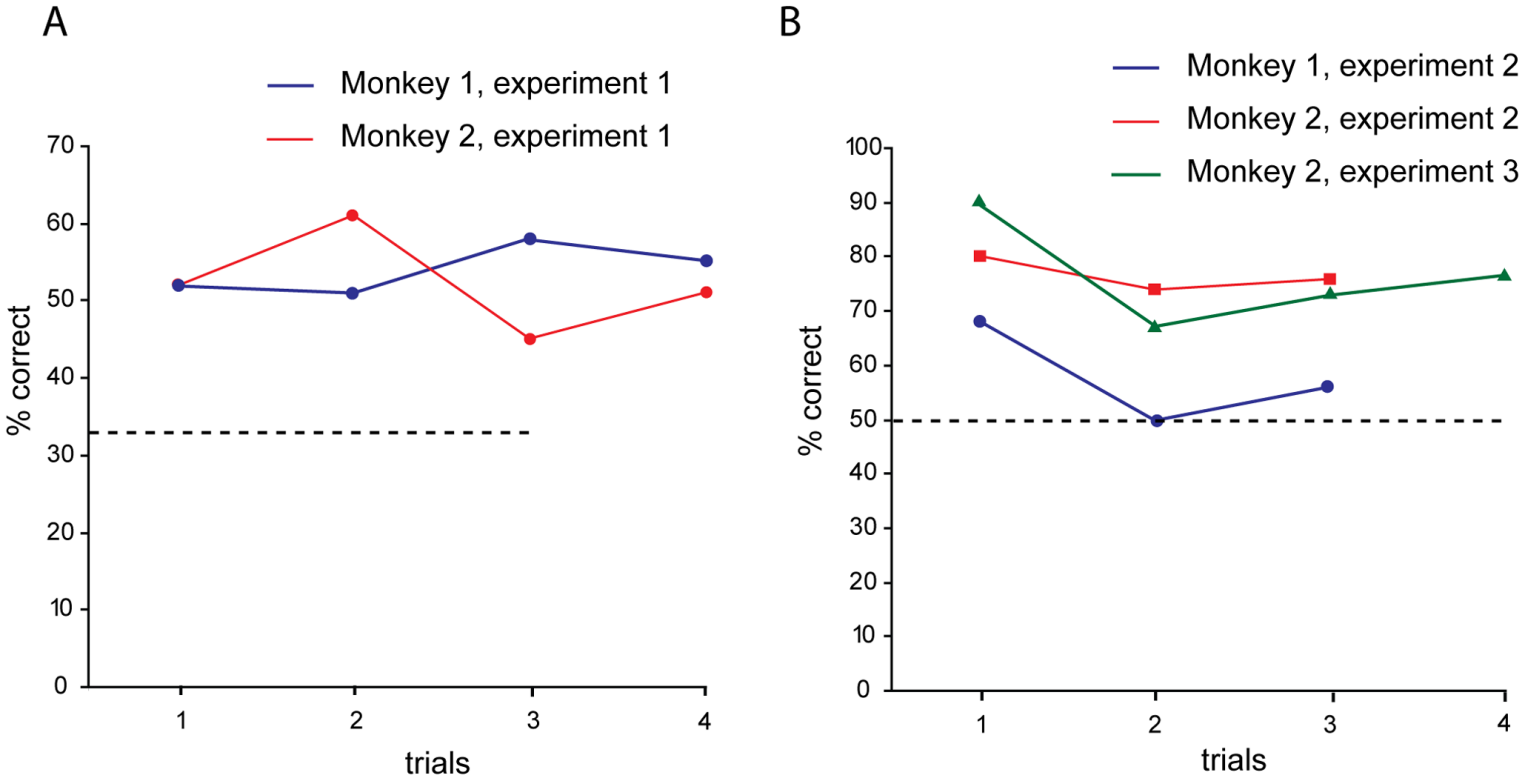
Performance as a function of the trial number during the test phase. **A.** Performance in experiment 2 with three tokens (blue line: monkey 1; red line: monkey 2). **B.** Performance with two tokens in experiment 2 with unequal presentation (blue line: monkey 1; red line: monkey 2) and experiment 3 (green line: monkey 2) with equal presentation. Dotted line: chance level.

First, we show the performance of the two monkeys taking into account only the first trial of the testing phase of all sessions. The choice of the monkey on its first testing trial, in fact, cannot be affected by any individual learning effect. Figure 2A shows the results for both monkeys. Both monkeys chose the valuable token in the first trial with a performance of 52% correct (16/31). Both scores were significantly different than the chance level of 33% (binomial test: p<0.05). We report also results of exchanges in the other trials (trial 2, 3 and 4) of the testing phase, although they might reflect information gained by both observation learning and individual learning. In addition, the result might reflect exploratory behavior of the alternative choices. Performance in the second, third and fourth trials were, respectively, at 51% (16/31), 58% (18/31) and 55% (17/31) in monkey 1, and at 61% (19/31), 45% (14/31) and 51% (16/31) in monkey 2. We also calculated the performance of the monkeys after the first correct choice, regardless if it was carried out in the first or the second trial. The percentage of correct choices in the second token exchange, after a correct first choice, was 58% (14/24) for monkey 1 and 76% (16/21) for monkey 2. Monkey 1 did not show a tendency to improve its performance in the trials following an individual success relative to the first trial immediately following the observation.

### Experiment 2

A similar analysis was performed on the results of the experiment 2. We calculated the performance of the two monkeys on the first trial in all the sessions.

Monkey 1 performed its first choice at 68% (34/50) and monkey 2 at 80% (40/50). Both scores were significantly different from chance (binomial test: p<0.05 in Monkey 1; p<0.001 in Monkey 2). As done for the three tokens task, we calculated the performance in the second and third trials, which were respectively 50% (25/50) and 56% (28/50) for monkey 1, and 74% (37/50) and 76% (38/50) for monkey 2. The percentage of correct choices for the second token exchange following a correct first choice was 38% (18/47) for monkey 1 and 73% (33/45) for monkey 2. Finally, we calculated the performance for the second trial and compared it to the accuracy of the first trial. After having performed correctly in the first trial, monkey 1 made a correct choice 16/33 times (48%) and an error 17/33 times; monkey 2 performed correctly 33/40 times (82%) and made an error 7/40 times.

After having made an error in the first trial, monkey 1 made an error again in the second trial 5/17 times (29%) and made a correct choice 12/17 times; monkey 2 made a subsequent error 6/10 times (60%) and chose the correct token 4/10 times in the second trial. Results from the second experiment show that monkey 2 was more skilled in this task than monkey 1, at least in a two token paradigm.

The lower level of performance of monkey 1 in the first trial after observation might not be related to a greater difficulty in learning by observation, but rather, could depend on a more general difficulty in the token exchange paradigm, or on an excessive explorative behavior. In fact, we noticed that this monkey showed difficulty in choosing the correct token even after its first correct choice. Having exchanged the correct token just before, the animal continued to make mistakes despite having directly experienced the token value. Performance in the second exchange after the first correct choice was in 58% with three tokens and 38% with two tokens. This exploratory behavior can also be seen by analyzing the data of the second trial. Animal 2 showed a more stable behavior by continuing to choose the object exchanged in the first trial (82% of correct answers after the first correct choice). Animal 1 preferred to change its first choice and explore other objects, with only 48% of correct response after a correct choice in the first trial.

### Experiment 3

We tested monkey 2 in the equal presentation version of the paradigm of the experiment 2 to control for the *stimulus enhancement* effect. We measured the performance of monkey 2 on the first trial of all the sessions after observation. The monkey performed its first choice at 90% (27/30), significantly different from chance (binomial test p<0.001). The performance in the second, third and fourth trials dropped a little to 67% (20/30), 73% (22/30) and 76% (23/30), respectively. Even limiting the analysis only to the results of the second exchange after the first successfully exchanged token in each session, the performance was not as high as for the first token exchanged after observation, being at 68% (20/29). We interpret the decrease in performance as the expression of an exploratory behavior of the potential alternatives. We predict that exploratory behavior would be reduced by a longer training period.

## Discussion

Several studies have shown that monkeys are able to interact socially and efficiently with humans, either by copying human facial movements when they are infants [22] or by matching the experimenter gestures such as clapping hands [23]. At a more abstract level, monkeys can also monitor the humans’ past goals in nonmatch-to-goal task [19], recognize that they are being imitated by humans [24], understand what humans see [25] and learn by observing simple discrimination learning problems [15]. Our study extends these results to show that monkeys are able to learn a token’s value by observing humans exchanging tokens.

Both macaque and capuchin monkeys can learn the “rules of token exchange” from humans [26], [27], [3], [4], [28], [9]. “Rules of token exchange” in the context of the present study are: 1) collecting an object from the metal tray and placing it into the palm of the experimenter’s hand to receive a reward, 2) only one token of each set is a valuable object and 3) the value of a token never changes. In the context of token exchange, monkeys are undoubtedly able to interact efficiently with humans.

Interaction is apparently less efficient when the token exchange takes place between conspecifics. Pele et al. [9] have shown that monkeys do not spontaneously exchange tokens with each other. “Token exchange” requires substantial training that cannot be done between naive animals. Humans are special partners for exchanging tokens because they can help control the training of the monkey. Further, we have shown that humans can contribute to training by being observed, even though an earlier study was unable to demonstrate monkeys learning by observation of humans [4]. We reconcile our results with this previous work by emphasizing the importance of vicarious reward. In the study of Brosnan and de Waal [4], vicarious reward was present when the monkey observed a conspecific and learned the value of a token. Vicarious reward was absent when the monkey observed a human and failed to learn the value of a token.

Observation learning has a number of potential advantages. It can inform the observer about the consequences of a behavior by substituting for direct feedback resulting from an action [29], [30] and may also contribute to cooperation and altruism [31]. Although it has been studied mostly in primates, vicarious learning occurs in other species, such as mice. For example, Jeon et al. [32] demonstrated that observing another mouse receive shocks is sufficient to develop fear conditioning in the observer.

The result of our first experiment showed that monkeys are able to learn token value by observing the humans exchange tokens in a three choice problem, albeit with modest performance. The second experiment showed that reducing the number of tokens does not dramatically improve performance as we had initially hypothesized. We found substantial individual differences between the two animals, especially in the second experiment. Further studies are clearly required to asses furthermore the interindividual variability in this task. Even so, in that experiment the performance of monkey 1 in the first trial after observation did not exceeded that exhibited after its first success. This indicates a general difficulty in learning token value, both through observation and through experience. In the third experiment, we tested whether unequal presentations of tokens contributed to a *stimulus enhancement* effect [17], whereby an association is created in the observer simply because an agent draws attention to an object (or location). The third experiment showed that by equally presenting valuable and neutral tokens, the monkey’s performance was as good as in the unequal paradigm adopted in the second experiment. Therefore, the results of the previous experiment cannot be explained in terms of *stimulus enhancement* produced by an unequal presentation of tokens.

In conclusion, our results are in line with the previous experiments in the literature, showing that monkeys can interact socially with humans, efficiently. We have identified the importance of vicarious reward and thus removed an important barrier to monkey observational learning using human models. This understanding opens opportunities for both comparative psychology and social neuroscience. We encourage broader adoption of human-monkey (H-M) paradigms of social interaction to supplement the growing use of monkey-monkey (M-M) interaction paradigms [33], [20]. M-M paradigms have weaknesses, like long or unfeasible training periods, that are not present in H-M paradigms. Along these lines, future behavioral studies could test the ability of monkeys to exchange self-valued and partner-valued tokens, akin to Pelé et al. [9]. Using a human for the monkey’s partner could speed training, especially in reciprocal tasks, permit greater experimental control and offer a broader range of experimental opportunities.
